# A comparison of telehealth versus in‐person group therapy: Results from a DBT‐based dual diagnosis IOP

**DOI:** 10.1002/jclp.23374

**Published:** 2022-05-09

**Authors:** Christian A. L. Bean, Pallavi Aurora, Corey J. Maddox, Ryan Mekota, Alanna Updegraff

**Affiliations:** ^1^ Department of Psychological Sciences Kent State University Kent Ohio USA; ^2^ Private Practice Mentor Ohio USA

**Keywords:** anxiety, depression, Dialectical Behavior Therapy, intensive outpatient program, Telehealth, videoconference

## Abstract

**Objective:**

The effectiveness of remotely delivered group interventions and treatments for individuals with more complex psychiatric presentations is understudied. Nevertheless, the emergence of the COVID‐19 pandemic shifted such treatments from in‐person to remote service delivery without the establishment of comparable effectiveness between in‐person and remote delivery. The current study presents the results of a private practice's transition from in‐person treatment delivery to a videoconference‐delivered Dialectical Behavior Therapy (DBT)‐based intensive outpatient program (IOP) for individuals with comorbid mental health and substance use disorder diagnoses in response to the pandemic.

**Methods:**

Change in symptoms of depression, anxiety, and stress following completion of the IOP was compared between the in‐person and videoconference groups.

**Results:**

Large reductions in symptoms were found following completion of the IOP for both the in‐person and videoconference groups. Furthermore, no significant differences in symptom reduction were found between the groups.

**Conclusion:**

Although large‐scale replication is needed, these results suggest that IOPs and other intensive group therapies delivered via videoconference may be as effective as in‐person therapies, even among individuals with more complex psychiatric presentations. Providers who have transitioned group therapies to videoconference formats or are considering creating remote groups can be more confident that they are not sacrificing treatment efficacy.

## 1 INTRODUCTION

In late 2019, a novel coronavirus (severe acute respiratory syndrome coronavirus 2; SARS‐CoV‐2), commonly known as COVID‐19, emerged and spread worldwide (Esakandari et al., 2020). The spread of COVID‐19 led to significant mortality and morbidity worldwide, subsequently increasing levels of fear, anxiety, and distress (Brooks et al., 2020). To reduce the spread of the COVID‐19 virus, social distancing protocols involving increased distance between people and reduced physical contact with others were widely recommended (Price & van Holm, 2021), necessitating a transition to remote interactions whenever possible. Indeed, these recommendations were also applied to psychological and physical health appointments. In swift efforts to mitigate the loss of treatment access, a wide‐scale transition to remotely delivered psychotherapy appointments was employed, informed by the knowledge that large‐scale disasters tend to exacerbate existing psychological symptoms and increase vulnerability to new onsets of mental health disorders (Neria et al., 2008; Nolen‐Hoeksema & Morrow, 1991). The transition from in‐person to remotely delivered services around the world necessitated by the pandemic outpaced the science, as studies examining the effectiveness of telehealth interventions are limited, especially in group therapy formats. Thus, the present study sought to fill this gap in the literature by comparing the effectiveness of a Dialectical Behavior Therapy (DBT)‐based intensive outpatient group program for patients seeking treatment for co‐occurring mental health and substance use disorder diagnoses delivered in‐person and remotely via videoconference.

### 1.1 Unpacking telehealth and telepsychology

Telepsychology, a subset of telehealth services, has been defined as “the provision of psychological services using telecommunication technologies” (Joint Task Force for the Development of Telepsychology Guidelines for Psychologists, 2013, p. 791). These technologies include, but are not limited to, telephones, smartphones, and videoconference platforms. The potential benefits of delivering psychotherapy using remote technology are numerous, including elimination of a transportation burden, greater access to care, decreased no‐show rates, reduced time commitment, and diminished need for childcare (Leach & Christensen, 2006; Reay et al., 2020). Furthermore, the effectiveness of telepsychological interventions has been demonstrated across a variety of patient populations, diagnoses (e.g., anxiety, depression, and substance use disorders), and treatment settings (Gentry et al., 2019). However, despite the significant benefits telepsychological services can offer patients, the availability and use of such services were limited before the onset of the COVID‐19 pandemic (Reay et al., 2020).

Researchers have examined patient satisfaction with telepsychological services as well as the effectiveness of such services, specifically treatments delivered via telephone or videoconference, for several Cognitive Behavioral Therapy (CBT)‐based treatments. Broadly, researchers have found high patient satisfaction with telepsychology before and during the COVID‐19 pandemic (Skime et al., 2022; Sugarman et al., 2021). Furthermore, in two studies by Mohr et al. (2006, 2012), researchers compared the use of telephone‐delivered CBT for depression to in‐person treatment. Both studies found significant improvements in depressive symptoms for both in‐person and telephone‐delivered CBT. Similar results were observed by Stubbings et al. (2013); who found no significant differences between videoconference‐delivered CBT and in‐person CBT, with participants in both conditions showing significant reductions in symptoms of depression, anxiety, and stress. Comparable effects for CBT treatments delivered via telepsychology and in‐person have also been found for patients with obsessive‐compulsive disorder (Lovell et al., 2006; Vogel et al., 2014) and posttraumatic stress disorder (PTSD) (Varker et al., 2019; Yuen et al., 2015).

The unprecedented, widespread transition to telepsychology during the COVID‐19 pandemic necessitated an increased examination of the effectiveness and acceptability of remotely delivered group treatment formats such as intensive outpatient programs (IOPs), which feature frequent and intensive group meetings. Current research suggests that group treatments delivering evidence‐based treatments via telepsychology enable increased flexibility and continuation of care (Childs et al., 2020). In a study conducted by Skime et al. (2022), patients with severe mental illness receiving CBT‐based treatments (i.e., behavioral activation, DBT, and Acceptance and Commitment Therapy) reported high levels of satisfaction, comfort, and convenience. Some patients also reported that telepsychology platforms facilitated increased social support during the COVID‐19 pandemic. Additionally, preliminary evidence regarding the effectiveness of remotely delivered IOPs has been provided by Puspitasari et al. (2021), who found that patients completing a videoconference‐delivered IOP demonstrated significant reductions in depression and anxiety.

Although there are several clear benefits of telepsychologically delivered treatments, there are some important limitations and potential risks to consider. For instance, it may be more difficult to ensure privacy and confidentiality for both patients and providers, engage in certain treatments such as exposure therapy (Appleton et al., 2021), and address physical health aspects of treatment (e.g., assess for symptoms of withdrawal, conduct drug screens, etc.). Furthermore, technological issues may be a potential barrier for patients. It is also important to consider provider competencies for delivering treatments via telepsychology to ensure patients receive the best possible treatment experience. One key competency necessary for providers is an understanding of geographical barriers outlined by licensing boards. It is imperative for providers to practice within their jurisdiction to ensure ethical care. Additional competencies for providers include understanding security and privacy measures needed to maintain confidentiality (e.g., secure communication methods), assessing appropriateness for treatment and the necessary level of care, modifying delivery of treatments, and understanding how telehealth may affect insurance/payment options (for a full review of provider competencies see Martin et al., 2020). Although these extra competencies might pose an initial barrier to treatment provision, there are several webinar and training opportunities currently being offered to fill this gap (Martin et al., 2020). Thus, although there are limitations to its use, the shift to telepsychological treatments during the COVID‐19 pandemic has been found to be effective in providing access to care and continuation of treatment (Appleton et al., 2021).

### 1.2 Dual diagnosis and telehealth

Research examining the effectiveness of telepsychology in the treatment of more complicated psychiatric presentations is limited, particularly among individuals who are dually diagnosed with comorbid mental health and substance use disorders. The prevalence of substance use disorders has dramatically increased over the past decade (Lin et al., 2019), yet access and utilization of effective treatments remain limited (Cummings et al., 2014; Park‐Lee et al., 2017). Indeed, these limitations are largely due to the unique challenges associated with treating patients with substance use concerns. To address these challenges, telepsychological interventions for substance use disorders have received increased attention. In a recent review, Lin et al. (2019) examined substance use treatments remotely delivered via videoconference. Their findings suggest that videoconference‐delivered interventions were effective treatment options for patients with substance use disorders. Additionally, patients also tended to report increased satisfaction with treatment. Although this review begins to highlight the advantages of utilizing telehealth services for substance use patients, the studies reviewed were limited in their methodological procedures as well as comparison groups. Thus, further examination is necessary to determine the effectiveness of remotely delivered treatments for patients with substance use concerns.

Given the high comorbidity rates between substance use disorders and many other mental health disorders, including mood, anxiety, personality, and trauma‐related disorders (Grant et al., 2015; Harris & Edlund, 2005), researchers have advocated for increased adoption of an integrated dual diagnosis approach (Buckner et al., 2008; Spivak et al., 2020). Specifically, this approach highlights the dual nature of substance use disorders co‐occurring with another mental health disorder, with the goal of reducing symptoms of both disorders concurrently, rather than only focusing on each diagnosis separately and in isolation. Indeed, research has found that integrated dual diagnosis treatments can be effectively delivered via telepsychology. For example, in a review by Gilmore et al. (2017) examining technology‐based interventions for co‐occurring substance use and trauma‐related symptoms, researchers generally found reductions in both trauma‐related and substance use symptoms, highlighting the feasibility and effectiveness of telepsychological services for dual diagnosis treatment. Furthermore, in a study conducted by Sugarman et al. (2021) during the COVID‐19 pandemic, patients receiving telepsychological treatment for dual diagnosis reported being “very satisfied” for both individual (90%) and group (58%) treatments approaches. Taken together, telepsychological treatments have been shown to be effective in reducing symptoms and increasing patient treatment satisfaction and attendance for individuals with substance use disorders and other comorbid diagnoses. However, there is a lack of research comparing in‐person and videoconference‐based intensive treatments for dual diagnosis patients delivered in a group format, which the present study sought to accomplish.

### 1.3 Current investigation

The present study sought to compare the delivery of in‐person versus videoconference‐based DBT treatment for patients enrolled in a dual diagnosis IOP. During the onset of the COVID‐19 pandemic, many treatment providers transitioned to remote intervention to accommodate recommended physical distance guidelines. This transition provided opportunities to leverage technology to provide treatment for patients with substance use disorders (McDonnell et al., 2021). Furthermore, along with traditional CBT treatments, many providers also found utilizing several third‐wave treatments such as DBT helpful in promoting improved quality of life and building skills of distress tolerance, mindfulness, emotion regulation, and interpersonal effectiveness (Hyland et al., 2022; Zalewski et al., 2021). Although there is a growing body of research examining the benefits of telepsychological services, there is limited research examining the use of telehealth services in group or IOP formats with patients seeking dual diagnosis treatment. Thus, the present study sought to fill this gap in the literature by examining potential differences in overall symptom reduction between in‐person and videoconference‐delivered treatment following completion of a DBT‐based dual diagnosis IOP.

## 2 METHOD

### 2.1 Participants and procedure

Eligible participants for this study were all patients who completed the dual diagnosis IOP at a suburban private practice located in Northeast Ohio from January 2018 through June 2021. During this time period, 96 patients completed the program, 69 of whom provided complete symptom assessment data and thus make up the final study sample. Patients engaged in either an in‐person or remote, videoconference‐delivered format of the IOP. Patients were transitioned to a videoconference‐delivered format (via Zoom) due to the COVID‐19 pandemic on March 21, 2020. As such, all patients who completed the program before the transition date were considered part of the in‐person group (*n* = 49), while those completing the program afterward were members of the videoconference group (*n* = 20).

All patients reported demographic information and prior treatment history at the initial intake session. Following this intake session, patients referred to the dual diagnosis IOP completed an hour‐long individual orientation session with one of the treatment team members to discuss program structure and rules and process any patient concerns or apprehensions. The orientation session was identical for the videoconference group except it was also used as an opportunity to troubleshoot any patient difficulties utilizing the videoconference platform used for the program. Participants in both groups were also asked to complete a self‐report measure to assess symptoms of depression, anxiety, and stress immediately before beginning the program as part of the usual orientation procedure. Patients were requested to complete this measure a second time upon program completion as part of the graduation packet they received at the end of their final session. Change in symptom scores following program completion was assessed for each subgroup. Patients did not receive compensation for providing data and, as part of the informed consent process, were assured that the decision to do so was voluntary and would not affect treatment in any way. Ethics approval was provided by the affiliated university's Institutional Review Board.

### 2.2 Symptom measure 

The Depression Anxiety Stress Scales (DASS; Lovibond & Lovibond, 1995) is a 42‐item self‐report measure comprised of three subscales examining symptoms of depression, anxiety, and stress. The DASS asks patients to indicate how much each item applied to them over the past week on a scale of 0 (did not apply to me at all) to 3 (applied to me very much, or most of the time). Each subscale includes 14 unique items. Possible scores on each subscale thus range from 0 to 42, with greater total scores indicating greater symptomology. Internal consistency estimates in this sample were not able to be computed due to the lack of item‐level data for some patients. However, previous research in clinical samples has shown the DASS to possess good test−retest reliability and excellent internal consistency, with subscale *α's* ranging from 0.88 to 0.96 (Brown et al., 1997; Page et al., 2007). Furthermore, the DASS has demonstrated strong convergent and discriminant validity with other established measures of anxiety and depressive symptoms as well as with diagnoses of anxiety and depressive disorders (Brown et al., 1997; Page et al., 2007). As noted earlier, the DASS was administered twice, first as part of the IOP orientation and again upon program completion.

### 2.3 IOP

All patients referred to the dual diagnosis IOP were allowed to participate, provided they had co‐occurring diagnoses of a substance use disorder and at least one mental health disorder (most commonly depression, anxiety, or PTSD). The IOP was conducted in an open‐group format, with group sizes ranging from approximately 6 to 12 people. Patients were able to join the group on a rolling basis. Therefore, the group consisted of new members and members who have already been engaging in treatment within the group. Approximately one new member joined per week. Patients were asked to complete three sessions per week for 8 weeks, with each session lasting 3 h. Thus, successful completion of the program consisted of attending at least 24 sessions coupled with consistent abstinence from all substance usage (except nicotine). Additional sessions were added at the conclusion of the 24‐session program, on an as‐needed basis, for individuals experiencing continued distress or elevated symptoms (e.g., severe suicidal ideation or difficulty maintaining abstinence). The dual diagnosis IOP utilized a DBT skills‐driven approach with a minimum of one new skill taught each session and discussion of diary cards, which patients used to track symptoms and maladaptive behaviors as well as use and practice of skills learned in group (Linehan, 2015). The IOP also incorporated elements specific to substance use disorders such as psychoeducation on the physiological effects of drugs and alcohol. Each session was led by a master's level therapist independently licensed in chemical dependency and co‐led by a master's level doctoral candidate in clinical psychology. The IOP was supervised by a licensed clinical psychologist through both weekly individual supervision with each leader separately and bi‐weekly group supervision. The group leaders and supervising psychologist completed American Psychological Association (APA) approved trainings in telepsychology to prepare for the transition of the IOP to a remote format at the beginning of the COVID‐19 pandemic.

### 2.4 Orientation session

As previously noted, participants engaged in an hour‐long informational orientation session before admission into the dual diagnosis IOP. During each orientation session, one of the group leaders reviewed group guidelines, including full abstinence from all substances and the attendance policy, and processed any potential patient concerns. Insurance and payment plans were also discussed. For individuals completing the IOP program via telehealth, the orientation session was an opportunity to troubleshoot the videoconference platform used.

### 2.5 IOP session procedure

Standard in‐person session protocol began with a review of the DBT skill introduced in the previous session for the first 30−45 min of the session. Following skill review, each patient reviewed their individual diary card by rating the intensity of their negative and positive emotional experiences and skills utilized since the previous IOP session. Subsequently, therapists provided feedback and engaged in brief therapy for approximately 5 min with each patient to ensure heightened stressors were addressed. Risk for suicidal and homicidal ideation was assessed each session. Following completion of diary card review, therapists introduced one new DBT skill to conclude the session. Upon completion of the full duration of the IOP, patients would have been exposed to nearly every DBT skill in the areas of core mindfulness, emotion regulation, distress tolerance, and interpersonal effectiveness (see Linehan, 2015 for more information on specific skills). Halfway through the session, all participants received a 15‐min break. Participants were able to utilize this break as they chose (e.g., eat, talk with other members, or go for a walk).

The sessions delivered via videoconference were modeled after the in‐person sessions as much as possible, with identical session structure and skill‐learning progressions. The use of the Zoom videoconference platform required handouts to be emailed to participants and for the co‐leader to spend time attending to patient issues with connectivity while the leader focused on skills teaching, although we found this fit nicely with the co‐leader's usual role of attending to attendance‐related issues (see Linehan, 2015 for a discussion of leadership roles in DBT skills groups). Patients were encouraged to turn off their cameras and mute their microphones during breaks unless they wished to speak with another group member. The videoconference platform seemed to lend itself well to recreating important aspects of the teaching experience with fidelity, such as Zoom's whiteboard feature substituting for standard whiteboards. Some features of Zoom, such as the ability for the leader and group members to easily share their screens to present completed worksheets to the entire group at once, represented enhancements over traditional, in‐person sessions. Participant identity and privacy were confirmed before each remote group session, with most participants joining from either computers or smartphones. To maintain patient confidentiality and reduce distractions, participants were asked to engage in IOP sessions in a quiet, private room, although this was more difficult for patients without childcare access (for a more detailed description of the experienced benefits and difficulties of delivering IOP over videoconference, please see Table 1).

**Table 1:** Comparison of benefits and risks between in‐person and videoconference‐delivered services.

In‐person treatment		Videoconference‐delivered treatment	
Benefits	Risks/limitations	Benefits	Risks/limitations
Face‐to‐face contact	Greater commuting time	Increased access to treatment	Requires access to internet/technology
Private sessions in secure office	Transportation required	Reduced or eliminated commuting time	Reduced privacy
Delivery of materials (e.g., whiteboard)	Sessions in office (limited by social distance recommendations)	Build social support during a pandemic	Potential for increased distractions (e.g., web surfing)
Effective and beneficial treatment	Decreased communication with providers outside session	Ability to have sessions at home	Potential technological issues (e.g., connectivity)
Build social support in group settings		No transportation necessary	Limited features on online platforms
Increased treatment options		Delivery of materials (e.g., share screen)	Requires development of additional competency
Enables in‐person screenings (e.g., drug screens)		Effective and beneficial treatment	More easily interrupted by others
Opportunity for full‐body hygiene and behavioral observation		Continuation of care during the pandemic	
		Extended platforms of communication (e.g., secure messaging)	

*Note*: This is not exhaustive list of benefits and risks, but an overview summary of commonly encountered benefits and risk. Information was gathered from our own experiences along with the following sources: Appleton et al. (2021), Hyland et al. (2022), Sugarman et al. (2021), and Skime et al. (2022).

## 3 RESULTS

### 3.1 Preliminary analyses

The overall study sample primarily identified as male (73.91%) and European American/White (94.20%), with the remaining patients identifying as African American/Black (2.90%) or Hispanic/Latino (1.45%). Information on racial/ethnic identity was missing for one patient. There were no significant differences between the in‐person and videoconference groups on any demographic or clinical variable at baseline (all *p'*s ≥ 0.09; see Table 2). There were also no significant differences observed in the number of sessions attended *t*(67) = 0.12, *p* = 0.90, or number of sessions missed for any reason, *t*(67) = 0.51, *p* = 0.61. Thus, no evidence was found for any difference in attendance between in‐person and videoconference‐delivered IOP.

**Table 2:** Clinical and demographic group characteristics at baseline.

	In‐person (*n* = 49)	Videoconference (*n* = 20)	*p *Value
Characteristic (*n* (%))			
Female	10 (20.41%)	8 (40.00%)	0.09
Race/ethnicity			0.23
European American/White	47 (95.92%)	18 (90.00%)	
African American/Black	1 (2.04%)	1 (5.00%)	
Hispanic/Latino	0	1 (5.00%)	
Unknown	1 (2.04%)	0	
Education			0.82
High school	14 (28.57%)	6 (30.00%)	
Some college	20 (40.82%)	6 (30.00%)	
Bachelor's degree	12 (24.49%)	6 (30.00%)	
Master's degree	3 (6.12%)	2 (10.00%)	
Employed	36 (73.47%)	16 (80.00%)	0.57
Married/cohabiting	28 (57.14%)	13 (65.00%)	0.55
On psychotropic medication	30 (61.22%)	14 (70.00%)	0.49
Prior psychological treatment	39 (81.25%)	14 (70.00%)	0.31
Characteristic (*M* (SD))			
Age	40.67 (12.71)	40.35 (10.70)	0.92
Sessions attended	25.98 (3.29)	26.10 (4.41)	0.90
Sessions missed	3.84 (4.05)	3.30 (3.87)	0.61

*Note*: Group differences were calculated using *χ* ^2^ tests of independence for categorical variables and independent‐samples *t*‐tests for continuous variables.

Abbreviation: SD, standard deviation.

Patients in the in‐person group who provided complete symptom data at both assessments did not differ on any clinical or demographic variable from those who did not (all *p'*s > 0.05). However, patients who provided all data in the videoconference group were significantly older (*M* = 40.35, standard deviation [SD] = 10.70) than graduates of the videoconference group who did not provide symptom data at both assessments (*M* = 32.42, SD = 13.03; *t*(37) = 2.08, *p* = 0.04). Additionally, a significant difference was observed in rates of symptom assessment completion (*χ* ^2^(1) = 12.11, *p* = 0.001), with a greater percentage of patients in the in‐person group providing complete data than patients in the videoconference group (87.72% vs. 56.41%, respectively).

### 3.2 Main analyses

We first examined whether significant change was observed in depression, anxiety, and stress within each group separately using a series of paired‐samples *t*‐tests. To adjust for multiple comparisons (as we examined three separate criteria for improvement), the conservative Bonferroni correction was used, setting the critical *p*‐value for statistical significance to 0.017. The in‐person IOP group demonstrated significant reductions in depression *t*(48) = 5.60, *p* < 0.001, Cohen's *d* = 0.80 (95% confidence interval [CI] = 0.47, 1.12), anxiety *t*(48) = 5.59, *p* < 0.001, Cohen's *d* = 0.80 (95% CI = 0.47, 1.12), and stress *t*(48) = 6.90, *p* < 0.001, Cohen's *d* = 0.99 (95% CI = 0.64, 1.32) from pre‐ to posttreatment with uniformly large effect sizes. Similarly, the videoconference IOP group showed significant reductions with large effect sizes in depression *t*(19 = 4.70, *p* < 0.001, Cohen's *d* = 1.05 (95% CI = 0.49, 1.59), anxiety *t*(19) = 3.99, *p* < 0.001, Cohen's *d* = 0.89 (95% CI = 0.36, 1.40), and stress *t*(19) = 3.61, *p* = 0.002, Cohen's *d* = 0.81 (95% CI = 0.29, 1.31) from pre‐ to posttreatment. Patients in both the in‐person and videoconference groups experienced significant improvement in psychological functioning after completing the dual diagnosis IOP as evidenced by large reductions in symptoms of depression, anxiety, and stress.

To determine whether the groups differed in the amount of change in symptoms over the course of treatment, and thus whether one mode of IOP delivery is relatively more effective, a series of mixed‐design repeated measures (2 × 2) analysis of variances were conducted, one for each DASS subscale. As the number of patients providing posttreatment data in the videoconference group was less than half that of the in‐person group, care was taken to ensure the assumption of equivalent error variance between groups was met so that the results of between‐group comparisons would be valid. Levene's test of equality of error variances was nonsignificant for all three DASS subscales at both baseline and posttreatment (all *p'*s > 0.17), indicating that equivalent error variances can be assumed. No significant time × group interaction was found for depression scores, *F*(1, 67) = 0.006, *p* = 0.94, $\eta_{p}^{2}$ < 0.001; see Table 3. Thus, it can be concluded that there was no significant difference between groups in the change of depression scores from pre‐ to posttreatment. Likewise, no significant time × group interactions were found for anxiety, *F*(1, 67) = 0.016, *p* = 0.90, $\eta_{p}^{2}$ < 0.001 or stress *F*(1, 67) = 0.059, *p* = 0.81, $\eta_{p}^{2}$ = 0.001. Taken together, the study results suggest that dual diagnosis IOP is effective in reducing symptoms of depression, anxiety, and stress when delivered in either in‐person or videoconference formats, with no significant difference between the forms of delivery in terms of symptom improvement.

**Table 3:** Changes in depression, anxiety, and stress scores following intensive outpatient therapy (IOP).

	In‐person group (*n* = 49)		Videoconference group (*n* = 20)			ANOVA			
	*M*	SD	*M*	SD	Effect	*F* ratio	*df*	*p*	$\eta_{p}^{2}$
DASS‐D									
Pretreatment	16.08	13.20	16.15	11.95	Time	41.91	1,67	<0.001	0.385
Posttreatment	4.37	7.16	4.15	4.76	Group	0.001	1,67	0.97	<0.001
					Time × group	0.006	1,67	0.94	<0.001
DASS‐A									
Pretreatment	11.63	10.45	12.15	10.23	Time	39.13	1,67	<0.001	0.369
Posttreatment	3.61	5.33	3.80	4.75	Group	0.041	1,67	0.84	0.001
					Time × group	0.016	1,67	0.90	<0.001
DASS‐S									
Pretreatment	18.14	12.27	20.45	12.95	Time	49.25	1,67	<0.001	0.424
Posttreatment	6.57	6.54	8.05	8.75	Group	0.815	1,67	0.37	0.012
					Time × group	0.059	1,67	0.81	0.001

Abbreviations: ANOVA, analysis of variance; DASS, Depression Anxiety Stress Scales; SD, standard deviation.

## 4 DISCUSSION

The current study sought to compare the efficacy of in‐person versus videoconference delivery of a DBT‐based IOP for patients with dual diagnoses. Both groups experienced large reductions in symptoms of depression, anxiety, and stress, with no significant differences in symptom improvement observed between the two groups. The videoconference‐delivered IOP was not only highly efficacious in reducing symptoms but produced results comparable to that of the in‐person IOP. These results suggest that videoconference can be an effective modality even for intensive delivery of psychotherapy in more complex clinical populations such as individuals with co‐occurring substance use disorders.

These results are timely given the ongoing effects of the COVID‐19 pandemic, in response to which many providers have already shifted therapeutic services to telehealth whenever possible. Relative to individual treatments, group therapies are particularly prone to cancellation or reductions in offered services required by social distancing guidelines. Remote delivery of groups, such as through videoconference, has therefore become more common and is likely to continue to increase in popularity. Our results, while preliminary, nevertheless support the efficacy of videoconference‐delivered intensive DBT‐based group therapy, building upon earlier findings demonstrating the effectiveness of telehealth in the delivery of individual CBT. Thus, providers who have moved their therapy groups to a videoconference‐delivered format or are considering doing so can have increased confidence that they are not sacrificing efficacy as a result. Nevertheless, treatment providers should seek appropriate training in the delivery of services via telehealth and associated technology as soon as possible to ensure they are able to uphold the highest standards of care and competently transfer their group programs to a remote format (see Martin et al., 2020).

It should be noted that the IOP completed by study participants was based on DBT skills along with elements specific to substance use disorders and did not include other aspects of comprehensive DBT such as intersession skills coaching and concurrent individual therapy (Linehan, 2015). DBT skills training as a primary, standalone intervention has been shown to be effective (Valentine et al., 2015), a view which is supported by our study. Still, there may be some instances in which more comprehensive DBT is indicated (see Mattei & Sposato, 2020 for a brief discussion). It will be important for future studies to determine whether any differences exist between comprehensive DBT delivered in‐person and remotely. For in‐depth discussions of challenges experienced in the process of, and suggestions for, translating both comprehensive DBT and DBT skills training to telehealth delivery, see Hyland et al. (2022), O'Hayer (2021), and Zalewski et al. (2021).

There are additional limitations to the study warranting mention. The study did not include long‐term follow‐ups on symptom measures beyond IOP completion nor was information on long‐term maintenance of sobriety available. It therefore remains for future studies to establish equivalence between in‐person and videoconference‐delivered IOPs in the maintenance of treatment gains. The sample size available, while comparable to other examinations of the effectiveness of telehealth‐delivered psychotherapy (e.g., Mohr et al., 2011; Lovell et al., 2006; Yuen et al., 2015), nevertheless renders the study results preliminary and in need of replication in large, preferably multisite, randomized controlled trials (RCTs). RCTs would also overcome the current study's lack of randomization, which limits the generalizability of results. While no significant differences in demographic, clinical, or attendance variables were found between groups, supporting group equivalency, we are nevertheless unable to determine if the general effects of the COVID‐19 pandemic impacted symptom trajectory during treatment, as the in‐person group received treatment before the onset of the pandemic. The possibility that the groups differed on some other unmeasured variable also cannot be ruled out. For instance, individuals with greater levels of technological savviness or comfort with technology may have been more likely to participate in and complete the videoconference‐delivered IOP. Alternatively, the in‐person IOP may have provided more opportunities for spontaneous socialization and so been relatively more beneficial for individuals who otherwise lacked such opportunities.

It will also be crucial for future studies to replicate these results in more demographically diverse samples, as the predominantly European‐American/White study sample significantly limits generalizability. In particular, previous research has shown that African American/Black and Hispanic identifying individuals are less likely to receive outpatient mental health care services compared to European‐American/White individuals (Cook et al., 2017). Additionally, racial/ethnic disparities may contribute to decreased willingness and ability to participate in IOPs delivered via telehealth (Childs et al., 2021). Furthermore, as the current study was conducted using treatment data from a private practice, it is important to consider that individuals from marginalized backgrounds may have less access to private substance use treatment programs and face additional barriers to treatment engagement (Matsuzaka & Knapp, 2020). Additional research is therefore urgently needed to determine if videoconference‐delivered IOPs are as effective as in‐person IOPs in diverse populations and across different treatment settings.

A significant strength of the current study is the collection of data as part of treatment as usual, lending ecological validity. As a result, however, patients were not incentivized to complete symptom assessments, which may have contributed to the relatively low rate of patients providing posttreatment data in the videoconference group. Graduates of the in‐person IOP were asked to complete the follow‐up assessment before leaving the building after their final session, while the questionnaire was emailed to graduates of the videoconference‐delivered IOP. Without incentivization, it was likely easier for graduates of the videoconference group to ignore emailed requests to complete the final assessment. While reasons patients chose not to complete the final assessment are unavailable, patients who provided complete symptom data did not differ on any clinical variable at baseline from those who did not, suggesting that the results may not be unduly biased due to the unprovided data. Finally, data were only available for individuals who completed the IOP, which may positively bias the effect size estimates of symptom reduction as a result of IOP participation. This would not be expected to affect the validity of comparisons between the in‐person and videoconference groups, however.

In sum, the current study provides preliminary evidence that intensive, videoconference‐delivered DBT‐based group therapies are not only effective in the treatment of depression and anxiety in patients with comorbid mental health and substance use concerns but may be as effective as traditional, in‐person treatment. Providers can therefore have increased confidence in the efficacy of intensive group therapies delivered via videoconference for the treatment of depression and anxiety.

## CONFLICTS OF INTEREST

The authors declare no conflicts of interest.

### 1 PEER REVIEW

The peer review history for this article is available at https://publons.com/publon/10.1002/jclp.23374

## Data Availability

The data that support the findings of this study are available on request from the corresponding author. The data are not publicly available due to privacy or ethical restrictions.
